# Viruses Associated with Ovarian Degeneration in *Apis mellifera* L. Queens

**DOI:** 10.1371/journal.pone.0016217

**Published:** 2011-01-25

**Authors:** Laurent Gauthier, Marc Ravallec, Magali Tournaire, François Cousserans, Max Bergoin, Benjamin Dainat, Joachim R. de Miranda

**Affiliations:** 1 Swiss Bee Research Centre, Agroscope Liebefeld-Posieux Research Station ALP, Bern, Switzerland; 2 Laboratoire de Biologie Intégrative et Virologie des Insectes, UMR 1231, Université Montpellier II, Montpellier, France; 3 Montpellier SupAgro, Montpellier, France; 4 School of Biological Sciences, Queen's University Belfast, Belfast, Northern Ireland; 5 Department of Ecology, Swedish University of Agricultural Sciences, Uppsala, Sweden; Montana State University, United States

## Abstract

Queen fecundity is a critical issue for the health of honeybee (*Apis mellifera* L.) colonies, as she is the only reproductive female in the colony and responsible for the constant renewal of the worker bee population. Any factor affecting the queen's fecundity will stagnate colony development, increasing its susceptibility to opportunistic pathogens. We discovered a pathology affecting the ovaries, characterized by a yellow discoloration concentrated in the apex of the ovaries resulting from degenerative lesions in the follicles. In extreme cases, marked by intense discoloration, the majority of the ovarioles were affected and these cases were universally associated with egg-laying deficiencies in the queens. Microscopic examination of the degenerated follicles showed extensive paracrystal lattices of 30 nm icosahedral viral particles. A cDNA library from degenerated ovaries contained a high frequency of deformed wing virus (DWV) and *Varroa destructor* virus 1 (VDV-1) sequences, two common and closely related honeybee Iflaviruses. These could also be identified by *in situ* hybridization in various parts of the ovary. A large-scale survey for 10 distinct honeybee viruses showed that DWV and VDV-1 were by far the most prevalent honeybee viruses in queen populations, with distinctly higher prevalence in mated queens (100% and 67%, respectively for DWV and VDV-1) than in virgin queens (37% and 0%, respectively). Since very high viral titres could be recorded in the ovaries and abdomens of both functional and deficient queens, no significant correlation could be made between viral titre and ovarian degeneration or egg-laying deficiency among the wider population of queens. Although our data suggest that DWV and VDV-1 have a role in extreme cases of ovarian degeneration, infection of the ovaries by these viruses does not necessarily result in ovarian degeneration, even at high titres, and additional factors are likely to be involved in this pathology.

## Introduction

The general decline in managed *Apis mellifera* L. colonies and beekeepers in Europe and the USA, as well as some other regions in the world, is a major challenge for ensuring adequate pollination of crops and wild plants [1]–[5]. Among the numerous possible causes involved in colony weakness and death, poor queen quality is often reported by beekeepers as a principal contributing factor [4]. This includes problems associated with low fecundity and untimely requeening events [6], [7].

For the honeybee (*Apis mellifera* L.), the queen is the sole reproductive female of the colony, producing a large number of sterile daughters (worker bees) who perform all the colony maintenance tasks, principally brood care, colony defence, construction and foraging. During the active season (spring to autumn in temperate climates), the worker bee population is constantly replaced, since the life span of the average adult bee is only a few weeks. In contrast, during the inactive season (winter in temperate climates), the queen reduces its egg-laying activity and winter bees can survive for months on the stored colony food reserves [8], [9]. The honeybee polyphenism (the separation of females into reproductive and non-reproductive castes) is determined by discrete changes during postembryonic development, commencing with the differential feeding of female larvae [10]. Larvae destined to become queens are fed with protein-rich glandular secretions (royal jelly) secreted by the nurse bees in much greater amounts and for the entire duration of larval development, while worker bee larvae receive this in much smaller amounts, and for just a few days before switching to a blend of sugars and royal jelly based diet [8]. The nutritional stimuli affect the TOR (target of rapamycin) regulatory pathway [11] and trigger an endocrine response manifested by an elevated juvenile hormone titre in queen larvae, compared to worker bee larvae [12].

These changes result in a marked difference between the two female castes in the number of ovarioles produced, which range between 180 and 200 per ovary in queens and between 2 and 12 per ovary in worker bees [13]. It has been shown that the high juvenile hormone titre in queen larvae prevents the induction of programmed cell death in the ovarioles at the onset of metamorphosis [12], [14]. This means that the majority of ovarioles degenerate in worker bees, while in queens these all survive and differentiate during development. Two regions can be distinguished in the ovariole: the upper filament and the germarium, where the follicles initiate and differentiate into oocytes and nurse cells, and the vitellarium, where the oocytes grow until they are released into the oviducts. In the vitellarium, each oocyte is associated to a group of sister cells whose function is to fill the oocyte with food reserves which will be used by the future embryo [15], [16]. Once the oocyte development is completed, the nurse cells degenerate. It is estimated that 3 to 5 follicles are produced every day in each ovariole [17]. Besides its role as an egg-laying machine, the queen releases different pheromone bouquets that maintain the social cohesion of the colony [18].

Despite the possible relevance for colony collapse events, there have been very few studies focusing on honeybee queen diseases. A major contributor to our knowledge of queen pathologies was W. Fyg who published in 1964 a report of his observations after he dissected thousands of queens in Switzerland [19]. Since then, it has been found that queens can be infected by several honeybee pathogens, including *Nosema sp.* and viruses [20]–[22]. Recently it has been shown that deformed wing virus (DWV), one of the most prevalent viruses infecting bee colonies, can be sexually transmitted by infected drones to the queen gonads and subsequently transovarially by the queen to her eggs [23]–[25]. However, no pathologies corresponding to such DWV infections in queens have so far been reported.

Here we report for the first time a pathology where numerous viral particles were found associated with degenerating ovariole follicles, which in extreme cases was furthermore also associated with severe fecundity problems of the affected queens. The only virus sequences recovered in abundance from a cDNA library of such degenerated follicles were for DWV and the closely related *Varroa destructor* virus 1 (VDV-1). *In situ* hybridization confirmed DWV and VDV-1 to be extensively distributed throughout the ovarian tissues, and hence the most likely identity of the virus particles in the degenerating follicles. However our data show that, despite their association with this pathology, these two viruses generally display a low virulence when infecting the queen reproductive organs, with little effect on queen function or fitness suggesting that other factors are most likely also involved in this pathology.

## Results

### 1. Design of the experiments

Two sets of experiments were conducted:

#### A: Survey of mated queens in France

We received a total of 130 mated queens (*Apis mellifera* L.) from French beekeepers between 2007 and 2009 (sampling A). The queens were either one, two or three years old, with roughly equal numbers of each age class. Most of the 1-year old queens were removed by beekeepers because of queen fecundity problems and weak development of the colony. The 2-year and 3-year old queens were generally removed by beekeepers as part of a regular management plan, independent of whether these displayed egg-laying deficiencies or not. Eighty eight queens were dissected and examined for the presence of internal pathologies. The tissues from all 130 queens were also processed for RNA extraction, in order to quantify the amount of deformed wing virus RNA using quantitative RT-qPCR. Among these 130 samples, a subset of 30 queens was analysed for the presence of 10 different bee viruses using classical qualitative RT-PCR assays.

For comparison, a sample of 40 virgin queens, reared in different apiaries in the South of France, were also assayed for these 10 bee viruses. Obviously, the egg-laying capacity of these queens is unknown.

#### B: Impact of DWV and VDV-1 infections on the queen fecundity

In this experiment, 59 2-year old mated queens were collected from a single apiary located in the South of France in 2008 (sampling B). All these queens had high fitness and fecundity characteristics and headed strong honey production colonies the previous year. Upon dissection, the ovaries were carefully checked for the presence of yellow discoloration symptoms. From this sample, 12 queens with yellowing ovaries and 12 queens with normal ovaries were chosen for detailed analysis, and their ovaries and abdomen were individually prepared for RNA extraction. We determined the presence of the 10 different bee viruses in these samples using qualitative RT-PCR (see above). Then, in order to look for possible impact of viral infection on ovary function, we determined by RT-qPCR the amounts of DWV and VDV-1 genomic RNA as well as the relative mRNA expression of two genes relevant to queen ovary health and function (vitellogenin and the vitellogenin receptor [26]), using β-actin as a ‘neutral’ internal reference gene for data normalization [27], and compared the relative expression in the ovaries with that for the corresponding abdomens.

### 2. Impaired queen egg-laying associated with degenerating ovarioles

**Figure 1:**
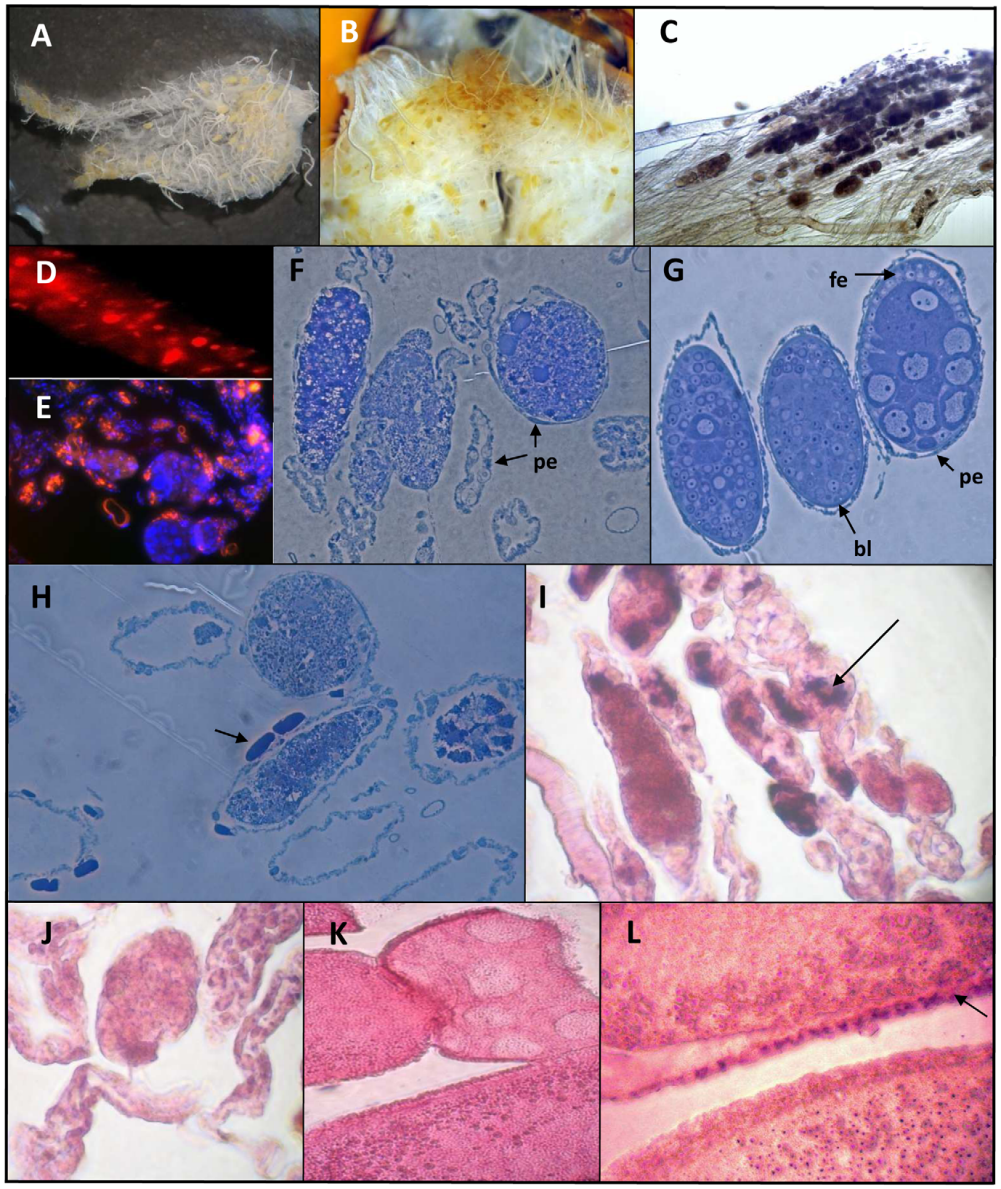
Histological analysis of the queen ovary. A and B: Stereomicroscopic observation of ovaries displaying yellowish colorations in the ovarioles. C: Clumps in the terminal filament of ovarioles observed by phase contrast microscopy (Olympus MVX 10). D: Propidium iodide staining of an ovariole showing nuclear condensation in the terminal filament (×600). E: general view of the apex of an ovary double stained with propidium iodide (red) and DAPI (blue). F, G and H: Toluidin blue staining of 1 µm thick cuttings from the germinal region of two ovaries. F and H: ovarioles displaying degeneration process showing detail of the disorganization of follicles surrounded by several empty ovarioles; H: presence of crystalline arrays in the peritoneal epithelium (arrow). G, normal ovary: detail of three follicles at different developmental stages. The nurse cells chamber and the oocyte chamber differentiation appear on the follicle located on the right. fe: follicular epithelium; pe: periteoneal epithelium; bl: basal lamina. I–L: In situ hybridization assays performed from paraffin embedded tissues for the detection of DWV/VDV-1 RNA. I and J: staining performed from the terminal filament region of an ovary using either antisense (I) or sense (J) probe. K and L: *in situ* analysis performed from the vitellarium showing DWV/VDV-1 RNA staining in the periphery of an oocyte (Arrow) with the antisens probe (L); K: control sense probe. Slices were counterstained with eosin Y.

Of the 130 mated queens sent in by beekeepers (sampling A), 65% had egg-laying deficiencies according to the beekeepers' observations. Although egg-laying deficiency was found in all age classes, most of the 1-year old queens sent in by beekeepers had egg-laying deficiency, while most of the 2-year and 3-year old queens replaced on rotation were considered healthy (Table 1). From the 130 queens received, a random subset of 88 queens was chosen for dissection. About half of these (56%) displayed a yellowish coloration in the apical part of their ovaries (Table 1, Figure 1A and 1B). In most cases this coloration was limited to a few ovarioles located in the periphery of the ovary. The presence of yellow discoloration in itself was not significantly correlated with either egg-laying troubles or with the age of the queen (Table 2). However, when comparing young and older queens subclasses, the presence of this coloration was found to be significantly related to an impairment in egg-laying activity in queens older than two years (Yates Corrected Chi-Square; P = 0.009). Such a relationship was not observed for younger mated queens (Yates Corrected Chi-Square; P = 0.262). However, the implication that the relationship between ovary discoloration and egg-laying deficiency is also dependent on the age of the queen has to be treated with caution, since older queens were sampled by different criteria (natural turnover) than the young queens (egg-laying defects). Furthermore, there are many possible causes for low fecundity among young queens, not all necessarily related to the discoloration observed here. Nevertheless, the possibility of an age-dependent relationship between ovarian discoloration and queen fecundity problems has both practical and scientific value and should be investigated in more detail.

**Table 1:** Percentages of mated queens (sampling A) of different ages displaying coloration on their ovaries (ovary coloration) upon dissection or associated with egg-laying troubles (fecundity troubles) according to beekeeper's observations.

*Queen age*	1 year	2 years	3 years
***Ovary coloration***	42% *(N = 36)*	69% *(N = 39)*	54% *(N = 13)*
***Egg-laying deficiency***	89% *(N = 37)*	62% *(N = 44)*	8% *(N = 25)*

N: number of samples analysed.

**Table 2:** Records of the different statistical tests performed from four different crossed variables (sampling A).

	Queen age	Ovary coloration	Egg-laying deficiency	DWV titre
Queen age	*-*	*-*	*-*	-
Ovary coloration	*Pearson X^2^ = 5.95* *P = 0.051*	*-*	*-*	-
Egg-laying deficiency	*Pearson X^2^ = 49.97* *P<0.001*	*Pearson X^2^ = 0.12* *P = 0.732*	*-*	-
DWV titre	*Kruskal-Wallis: 6.39* *P = 0.094*	*Kruskal-Wallis: 5.09* *P = 0.165*	*Mann-Whitney U: 2.63* *P = 0.067*	-

Data were considered as significant if P<0.05.

**Figure 2:**
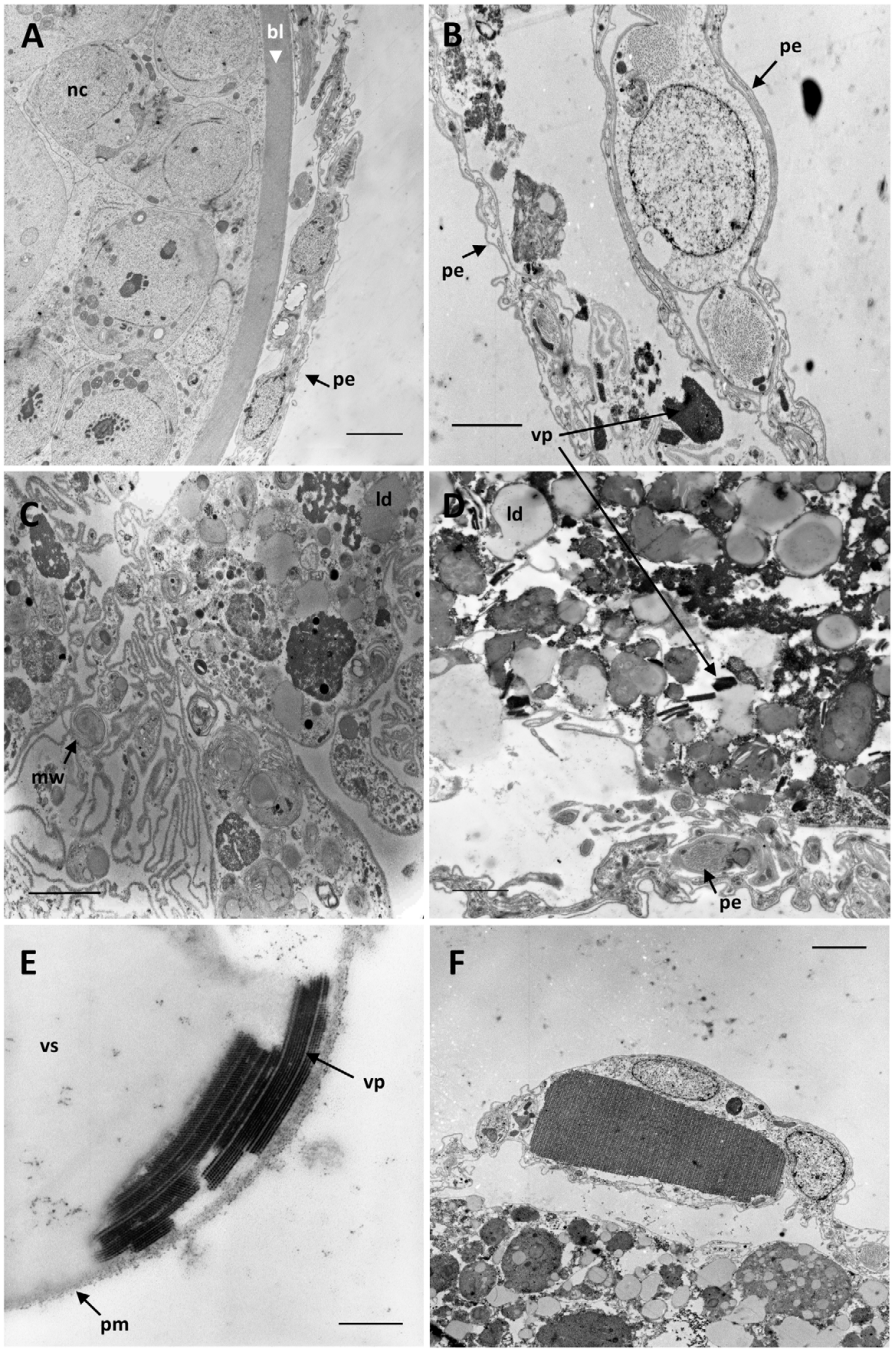
Ultrastructural observations in the germarium. A: TEM analysis of a normal follicle showing nurse cells (nc), the thick basal lamina (bl) and the peritoneal epithelium (pe). B: TEM analysis of an empty ovariole with presence of viral particles (vp) and cellular debris lined by the peritoneal enveloppe (pe). C and D: TEM analysis showing cellular degeneration of follicular cells with myelin whorls (mw), lipid droplets (ld), viral particles (vp) and peritoneal envelope (pe). E: TEM observation of viral particles (vp) and the virogenic stroma (vs) near the plasma membrane (pm) of a follicular cell. F: detail of a crystalline matrix in a peritoneal epithelial cell. Bars in electron micrographs represent 5 µm, 3 µm, 2 µm, 2 µm, 250 nm and 3 µm in figures A, B, C, D, E and F, respectively.

Among the 88 mated queens dissected from sampling A, 10 queens had an especially high degree of ovary coloration, with the majority of ovarioles displaying a yellow coloration which extended to the vitellarium part of the ovary (Figure 1B). These queens were all associated with egg-laying deficiency and had different ages (3 were less than 1 year old, 6 were 2 years old and 1 was 3 years old). In these queens, the ovaries were intensely coloured and appeared flat and empty when deposited on a glass slide (Figure 1A). In these, numerous masses of a coloured opaque material could be observed in the germinal part of the ovarioles as well as brownish colorations in some of the already formed oocytes located downstream (Figure 1B and 1C). Numerous dead cells were identified in these tissues by propidium iodide staining of individual ovarioles (Figure 1D and 1E). Classical histological and electron microscopy analysis revealed major lesions, characterized by cellular disorganization in the follicles, increasing intercellular spaces and the absence of the basal lamina in diseased tissues (Figure 1F and 1H, and Figure 2B) compared to healthy tissues (Figure 1G and Figure 2A). Four of these queens were also included in the complete virus survey (Table 3).

**Table 3:** Presence or absence of 10 different bee viruses in a sample of 30 mated queens collected from different regions of France (sampling A).

Sample	Troubles	Coloration	Age	Log10[DWV/bee]	DWV	VDV-1	BQCV	IAPV	SBV	ABPV	VdMLV	CBPV	KBV	SBPV
1	no	nd	3	9.9	**+**	**−**	**−**	**−**	**−**	**−**	**−**	**−**	**−**	**−**
2	no	nd	3	5.3	**+**	**−**	**−**	**−**	**−**	**−**	**−**	**−**	**−**	**−**
3	no	nd	2	9.7	**+**	**−**	**+**	**−**	**−**	**−**	**−**	**−**	**−**	**−**
4	no	nd	3	5.3	**+**	**+**	**+**	**+**	**−**	**−**	**−**	**−**	**−**	**−**
5	no	nd	3	5.5	**+**	**+**	**−**	**+**	**−**	**−**	**−**	**−**	**−**	**−**
6	no	nd	3	5.7	**+**	**−**	**−**	**−**	**−**	**−**	**−**	**−**	**−**	**−**
7	no	yes	3	6.8	**+**	**+**	**+**	**+**	**−**	**−**	**−**	**−**	**−**	**−**
8	no	yes	2	4.2	**+**	**−**	**−**	**−**	**−**	**−**	**−**	**−**	**−**	**−**
9	no	yes	2	6.7	**+**	**+**	**+**	**−**	**−**	**−**	**−**	**−**	**−**	**−**
10	no	yes	2	5.7	**+**	**+**	**+**	**−**	**−**	**−**	**−**	**−**	**−**	**−**
11	no	no	3	5.6	**+**	**+**	**+**	**−**	**−**	**−**	**−**	**−**	**−**	**−**
12	no	no	1	10.6	**+**	**+**	**−**	**−**	**−**	**−**	**−**	**−**	**−**	**−**
13	no	no	1	10.4	**+**	**+**	**+**	**−**	**−**	**−**	**−**	**−**	**−**	**−**
14	no	yes	2	6.8	**+**	**+**	**+**	**−**	**−**	**−**	**−**	**−**	**−**	**−**
15	no	no	1	5.8	**+**	**+**	**+**	**−**	**−**	**−**	**−**	**−**	**−**	**−**
16	no	yes	2	4.2	**+**	**+**	**−**	**−**	**−**	**−**	**−**	**−**	**−**	**−**
17	yes	nd	2	9.5	**+**	**−**	**−**	**−**	**−**	**−**	**−**	**−**	**−**	**−**
18	yes	no	1	6.1	**+**	**+**	**+**	**−**	**−**	**−**	**−**	**−**	**−**	**−**
19	yes	HIGH	1	8.9	**+**	**+**	**−**	**−**	**−**	**−**	**−**	**−**	**−**	**−**
20	yes	yes	1	9.6	**+**	**+**	**+**	**−**	**−**	**−**	**−**	**−**	**−**	**−**
21	yes	yes	1	4.9	**+**	**−**	**+**	**−**	**−**	**−**	**−**	**−**	**−**	**−**
22	yes	no	1	4.7	**+**	**+**	**+**	**−**	**−**	**−**	**−**	**−**	**−**	**−**
23	yes	HIGH	2	4.7	**+**	**+**	**+**	**−**	**−**	**−**	**−**	**−**	**−**	**−**
24	yes	HIGH	2	3.5	**+**	**+**	**−**	**−**	**−**	**−**	**−**	**−**	**−**	**−**
25	yes	yes	1	8.0	**+**	**+**	**−**	**−**	**−**	**−**	**−**	**−**	**−**	**−**
26	yes	yes	1	9.6	**+**	**−**	**+**	**−**	**−**	**−**	**−**	**−**	**−**	**−**
27	yes	no	1	3.0	**+**	**−**	**+**	**−**	**−**	**−**	**−**	**−**	**−**	**−**
28	yes	HIGH	1	8.6	**+**	**+**	**+**	**−**	**−**	**−**	**−**	**−**	**−**	**−**
29	yes	no	2	9.0	**+**	**+**	**+**	**−**	**−**	**−**	**−**	**−**	**−**	**−**
30	yes	yes	1	5.2	**+**	**−**	**+**	**−**	**+**	**−**	**−**	**−**	**−**	**−**
*%*					***100%***	***67%***	***63%***	***10%***	***3%***	***0%***	***0%***	***0%***	***0%***	***0%***

The respective age, fitness status (association with egg-laying troubles) and DWV titres recorded in each queen is indicated, as well as the presence of yellow colorations in the ovaries. nd: sample not dissected. HIGH: samples displaying a particular intense coloration in the ovary. ABPV: acute bee paralysis virus; VdMLV: *Varroa destructor* macula-like virus; BQCV: black queen cell virus; CBPV: chronic bee paralysis virus; DWV: deformed wing virus; IAPV: israeli acute bee paralysis virus; KBV: Kashmir bee virus; SBV: sacbrood virus; SBPV: slow paralysis virus; VDV-1: *Varroa destructor* virus-1.

### 3. Extensive viral paracrystalline arrays in degenerating ovariole follicles

Histological analysis of the germinal region of the intensely coloured ovaries revealed extensive paracrystalline lattices of viral particles in the follicular cells, either in the cytoplasm or near the cellular plasma membrane (Figure 2B, 2D and 2E). These viral paracrystalline lattices were generally arrayed with membrane-like complexes associated with myelin whorls, numerous lipid droplets and vacuoles (Figure 2C). In parallel, many crystalline inclusions could also often be observed in the peritoneal epithelium either by optical or by electron microscopy (Figure 1H and Figure 2F). However the crystal lattice of these latter inclusions was quite different from viral paracrystals observed in follicular cells (Figure 2E).

The location, shape and size of the virus particles, as well as their arrangement in paracrystalline arrays, is typical for insect picorna-like viruses, several of which infect honeybees. To determine the identity of the viruses arrayed in the degenerated ovaries we screened a cDNA library made from total RNA from degenerated queen ovaries. Twenty plasmids with cDNA inserts larger than 2 kb were sequenced. Five of these contained viral sequences, with three clones most closely related to VDV-1 (99% nucleotide identity) and the other two most closely related to DWV (98% nucleotide identity). All fragments were located in the non structural region of the DWV or VDV-1 genomes [28], [29], *i.e.* close to the poly-A tail that is naturally found at the 3′ end of the genome of this group of viruses [30]. The other fifteen clones contained *Apis mellifera* RNA sequences. This high frequency of DWV and VDV-1 sequences in the cDNA library suggests that these are most likely the viruses seen in the paracrystalline arrays in degenerated follicles.

This was partially confirmed by *in situ* hybridization studies, which detected large amounts of DWV/VDV-1 RNA in the germarium as well as in the outer envelope of some oocytes (Figure 1I and 1L). No hybridization was observed with either the sense or non sense control probes (Figure 1J and 1K respectively). However due to the lack of resolution of this method, we could not certify that the signal we obtained with the DWV/VDV-1 probe matched precisely with the viral paracrystalline lattices observed in follicles.

### 4. Prevalence of honeybee viruses in queens

**Figure 3:**
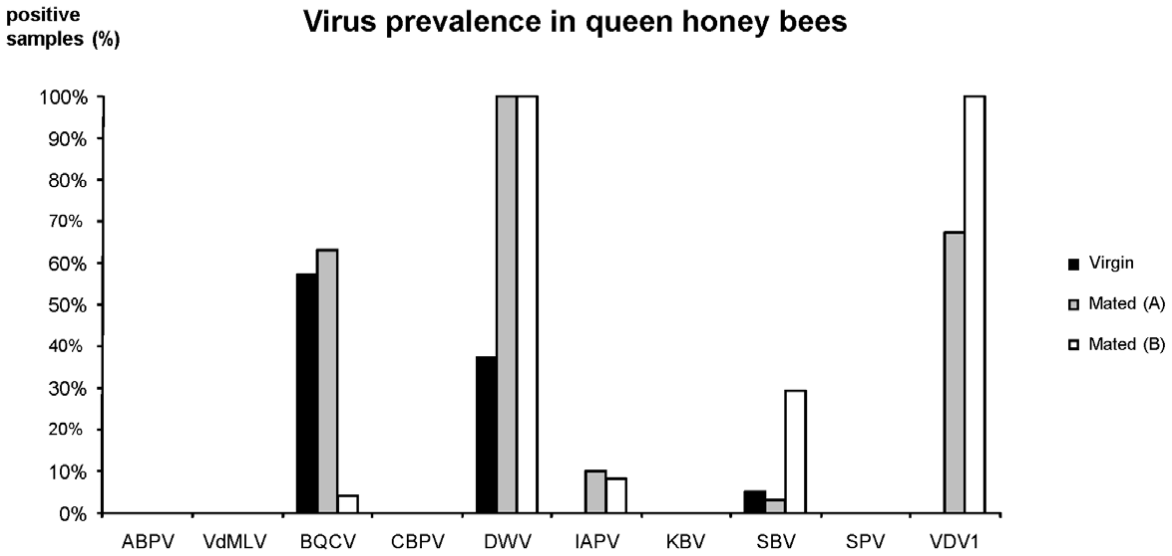
Detection of 10 bee viruses in virgin and mated queens. Mated queens were divided according to the 2 different samplings (A and B). Sampling A: sample made of 130 mated queens received from beekeepers. Sampling B: sample of 24 mated two year old queens displaying a high fitness. Virus abbreviations as in Table 3.

In order to further identify the viruses detected in queen ovaries by electron microscopy, we analysed a subset of the mated queen samples we received from various places in France (sampling A) for the presence or absence of 10 different honeybee viruses, using PCR-based assays. These data are presented in Table 3, together with relevant apicultural queen parameters. For comparison, we also analyzed 40 virgin queens from several apiaries and 24 healthy mated queens, half with ovary coloration and half without, collected in a single apiary in the South of France (sampling B; Figure 3). Only DWV (37%), sacbrood virus (SBV) (5%) and black queen cell virus (BQCV) (57%) were detected in the virgin queen samples. By contrast, five viruses were identified in the mated queens: DWV, SBV, BQCV, VDV-1 and Israeli acute paralysis virus (IAPV), with 83% of queens infected by more than one virus. However, the viruses were not equally distributed between the samplings A and B. Although DWV was detected in all the mated queens we analyzed, highly distinct prevalence values were recorded for SBV (3% and 29% in samplings A and B respectively); BQCV (63% and 4% in samplings A and B respectively); VDV-1 (67% and 100% in sampling A and B respectively) and IAPV (10% and 8% in samplings A and B respectively). *Varroa destructor* Macula-like virus (VdMLV), acute bee paralysis virus (ABPV), chronic bee paralysis virus (CBPV), slow bee paralysis virus (SBPV) and Kashmir bee virus (KBV) were not detected. We found no association between the qualitative detection of any of these viruses and either impaired egg-laying, queen age or the yellowing ovary symptoms, nor were there any associations among the different pathogens (not shown).

### 5. DWV titre in mated queen ovaries is unrelated to queen age, ovarian degeneration or fecundity problems

**Figure 4:**
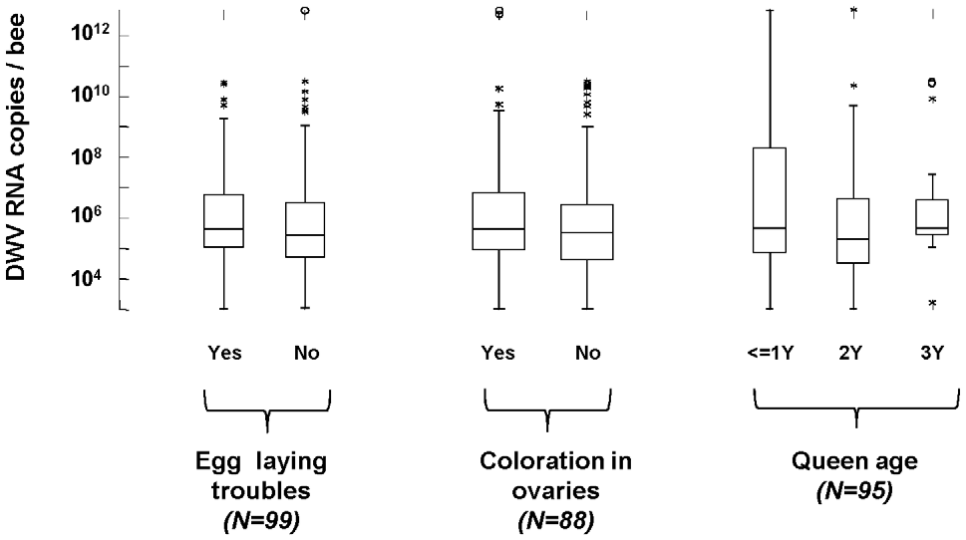
Distribution of DWV titres in queens samples (sampling A). Data are shown according to the presence of egg-laying troubles detected by beekeepers, the presence of yellow coloration in the upper ovary detected upon dissection and the queen age (Y = year). DWV quantitative values are indicated as equivalent genome copies deduced from a standard curve made of tenfold dilutions of a PCR fragment.

We used RT-qPCR to estimate the DWV titres in all the 130 mated queens analysed here and related these to their biological and pathological data. For these analyses, the entire queen was used. Although a very wide range of DWV titres was observed among these queens, there was no significant difference in the median DWV titre between queens of different age, between queens with or without fecundity problems or between queens with yellow coloration in their ovaries (Table 2 and Figure 4).

### 6. Pathogenicity of DWV and VDV-1 to honeybee queen ovaries

**Figure 5:**
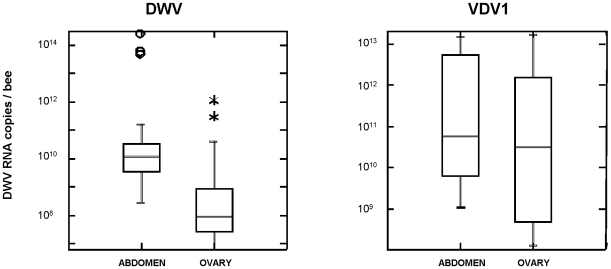
Distribution of DWV and VDV-1 loads in abdomen and ovaries of 24 queens samples (sampling B). DWV and VDV-1 quantitative values are indicated as equivalent genome copies deduced from standard curves made of tenfold dilutions of PCR fragments.

In order to study more precisely the influence of DWV and VDV-1 infections on queen physiology, we measured the expression of two genes, the vitellogenin and the vitellogenin receptor, that presumably could be affected by viral replication and tissue destruction and whose down-regulation would have a strong impact on queen fecundity. Because we could not link the presence of colorations with DWV titres nor with fertility problems observed by beekeepers in the 130 queens we first analysed, we also analysed 24 two year-old mated queens that never displayed any egg-laying defects and headed strong honey production colonies the previous year (sampling B). The queens were dissected and the ovaries and abdomens were processed separately for pathological, viral and cellular genes (vitellogenin and vitellogenin receptor) analyses. Half of these samples were chosen on the basis of yellow coloration on the apical part of their ovaries while the other half did not have such coloration. First we measured the respective amounts of DWV and VDV-1 RNA by quantitative RT-qPCR. Both viruses were detected in the abdomen of all these queens, but of the corresponding ovary samples, 4 contained neither DWV nor VDV-1, one contained only DWV, one only VDV-1 and the remaining 18 contained both viruses. As observed before, the virus titres varied considerably from one queen to another, covering a 10^6^ and 10^5^ fold range for DWV and VDV-1, respectively (Figure 5). Significantly higher VdV-1 than DWV titres were found in ovaries (P<0.01). The maximum titres we recorded in the ovaries of infected queens were about 10^12^ genome equivalent copies per ovary for DWV and 10^13^ genome equivalent copies for VDV-1. However our data showed a significant difference between abdomen and ovaries for both the DWV and VDV-1 titres (Mann-Whitney U test; P<0.001 and P<0.05, respectively), and a strong correlation between abdomen and ovary DWV titres (Pearson R = 0.988, P<0.001) but no such correlation was found for the VDV-1 titres (Pearson R = 0.293, P = 0.165) (see Figure S1). We found no correlation between the DWV and VDV-1 titres in either the abdomen (Pearson R = −0.093; P = 0.665) or the ovaries (Pearson R = −0.118; P = 0.583).

The expression of the vitellogenin gene (*Vg*) was higher in the abdomen than in the ovaries while the reverse was observed for the vitellogenin receptor gene (*Vg-R*) (see Figure S2). However we could not find any correlation between either the DWV or VDV-1 titres and the expression of either of these genes in the ovaries (Vitellogenin vs DWV and VDV-1: Pearson R = −0.017 and −0.210, respectively; Vitellogenin receptor vs DWV and VDV-1: Pearson R = 0.195 and −0.286, respectively) or in abdomen (Vitellogenin vs DWV and VDV-1: Pearson R = −0.101 and −0.220, respectively; Vitellogenin receptor vs DWV and VDV-1: Pearson R = −0.121 and −0.077, respectively).

## Discussion

The constant renewal of *Apis mellifera* worker bees is a prerequisite for maintaining the bee population throughout the year, and a strong, numerous adult bee population is essential for colony function, health and survival. Without an adequate adult bee population, essential colony tasks such as brood care, thermoregulation, defence and foraging are neglected. This invites the replication and spread of opportunistic pathogens in the colony, affecting brood or adults. The queen health and fecundity is therefore an important factor for colony health and survival, managed by natural colonies through supersedure of deficient queens and by beekeepers through regular queen replacement. Here we investigated the relationships between viral infections and a newly discovered clinical pathology of the ovaries, and assess the extent to which these affect queen health and function.

### Presence of viruses in queen ovaries associated to follicle degeneration

We report a new pathological condition of honeybee queens, affecting the germinal region of the ovarioles where follicles are differentiating. This condition consists of extensive lesions that appear at dissection level as masses of yellowish material in the germinal part of the ovaries. At microscopic level these lesions were found associated with large numbers of empty ovarioles. Electron microscopy studies revealed numerous 30 nm icosahedral viral particles in the germarium region of ovaries that were particularly severely affected by these symptoms. These viral particles were often assembled in paracrystalline arrays and accompanied by critical cellular disorganization symptoms, such as membrane-like complexes and myelin whorls which are typical of viral infections [31]. These paracrystals were sometimes so abundant that they could be distinguished on 1 µm sections observed under light microscopy. Molecular analyses of affected ovaries suggest that the viruses in these paracrystals are most likely deformed wing virus (DWV) and *Varroa destructor* virus-1 (VDV-1), two closely related, widespread and highly abundant honeybee viruses. The viral paracrystals were universally associated with the destruction of the basal lamina of the follicles but the peritoneal epithelium still remained remarkably intact. Other crystal formations that were also occasionally observed in the peritoneal epithelial cells are most likely due to protein accumulation from either bee or viral origin although we could not identify any associated viral particles in this tissue. Taken together, these observations suggest a resorption of the follicular cells, possibly induced by viral replication and particle accumulation. Programmed cell death via apoptosis or autophagy is an important mechanism for organ and tissue development and for the maintenance of tissue homeostasis and immunity, specially against viral infections [32], [33]. In *Apis mellifera* queen, this process was previously demonstrated in ovaries as a result of the hormonal imbalances that regulate caste differentiation [12]. In mated honeybee queens, cell death in the ovaries always occurs as a physiological consequence of oocyte maturation and ovulation; once the oocyte reaches its full maturation, the nurse cell chamber degenerate through an autophagic process. Pycnotic nuclei have also been observed in the germarium of virgin queen ovaries as well as in ovaries of workers kept in the presence of the queen, suggesting that apoptosis mechanism plays a major role in controlling follicle differentiation [34]. In our study, such cellular death patterns were evidenced by propidium iodide staining, both in the terminal filament and in the germinal part of the ovary (Figure 1D and 1E) but, according to their large distribution in tissues, may rather result from a pathological problem. Programmed cellular death seems also to be involved in oocyte resorption when environmental conditions are limiting the egg production of the queens [35]. However, the samples examined here were collected in spring during the warm season and consequently should not normally correspond to such environmentally induced oocyte resorption. These observations suggest that other factors could have triggered the follicle degeneration. Cases of ovarian atrophy corresponding to a rapid disintegration of the content of ovarioles and leading to the complete shrivelling of the ovary was also described by Fyg [19]. However these signs were always found associated with hypertrophy of the fat body with presence of a yellowish viscous hemolymph that we did not find here. Moreover Fyg never described yellowish coloration of the apex of the ovary, suggesting a different pathology.

Cell death in the honeybee queen ovaries may also be induced by different pathogens such as *Nosema sp.* infections which have been reported to be able to induce ovarian resorption. However these signs were found mostly initiated in the basal part of the ovary, in contrast to the condition we described here [36], [37]. Moreover we did not detect either *Nosema apis* or *Nosema ceranae* using PCR in the ovaries of the 24 queens suggesting that this pathogen is not directly involved in this pathology. During this survey we observed in rare cases melanisation processes or presence of nodules in the ovaries associated with fungal or bacterial infections, such as those described by Fyg [19]. However these cannot account for the degenerative symptoms we describe here (see Figure S3). Conversely the presence of extensive viral paracrystals in follicular cells is indicative of an intense viral replication and raises the question whether viral infections could also trigger such an autophagic process. Viral invasion of the terminal filament or the upper germarium where follicles progressively differentiate from stem cells can account for the ability of viruses to be transmitted vertically, as has been shown for a number of honeybee viruses, including DWV [23], [38]–[40]. Nevertheless very few viral particles should be involved in this process to avoid host responses such as apoptosis and the intense viral replication that we observed here is presumably not compatible with a regular vertical transmission pattern of viruses.

### Virus infections in queens

Although there are many viruses that can infect honeybees [20], [41], many were not detected in our queen samples. Here, the queens were found to be infected by DWV, VDV-1, BQCV, IAPV, and SBV, while the samples were negative for CBPV, ABPV, SPV, KBV and VdMLV. A similar virus survey performed in the USA from 30 mated queens collected in the same apiary showed the presence of DWV, BQCV, SBV, CBPV and KBV, but not ABPV [39]. Both surveys had a high incidence of co-infection (83% in our samples and 93% in USA), with DWV (100% in both surveys) and BQCV (63% in this survey and 86% in the USA) as the most prevalent viruses. Earlier studies from Australia using an immunological detection method also showed that BQCV was highly prevalent in queens [42]. Here we found however that BQCV prevalence was very variable according to the origin of the samples. Such apiary-specific differences were also observed for SBV. The prevalence of SBV in our study (3% and 29% in sampling A and B, respectively) was considerably lower than for the USA survey, where 62% of queen samples were found SBV positive [39]. Such large differences in virus prevalence according to geographic origin are also common for worker bee populations. For example, SBV prevalence was very low in Hungarian and United Kingdom surveys, relatively high in Austria and very high in Denmark and in France (2%, 1.4%, 49%, 81% and 86%, respectively). Likewise, BQCV prevalence in these surveys was 54%, 1.4%, 30%, 1% and 86%, respectively [43]–[46]. These contrasting results might be related to different local environmental conditions that may affect the transmission and epidemiology of the virus, to genetic polymorphism in viral genomes or to different host susceptibilities. From our data, the virus frequencies could not be associated with any deficiency in egg-laying or with the presence of primary pathology describe here, the degeneration of the ovarioles.

DWV and its close relative VDV-1 were found in a majority of the queens we examined, and at a range of titres. The result for DWV concurs with previous observations [24], [40], [47] and suggests that DWV at least is a common infection of honeybee queens. In our studies, VDV-1 was not detected in virgin queens. However, VDV-1 naturally has a far sparser prevalence and distribution than DWV in French worker bee populations, and is naturally more prevalent in Varroa mite populations than in the corresponding honeybee populations ([48]; de Miranda, Tournaire, Paxton and Gauthier, unpublished). We found large differences in DWV titres between mated queens of the same age suggesting that some individuals may be more permissive to DWV infection. Partly as a result of this large internal variation, no significant difference in the median DWV titre could be observed between queens of different ages. A more detailed analysis of 24 apparently healthy and fully functional queens of the same age showed a strong correlation between DWV titres in abdomen and ovaries but an absence of a similar correlation for VDV-1. The reason for this discrepancy is unknown. The virus titres were higher in abdomen than in the ovaries, suggesting that other tissues such as fat body and gut where this virus was previously identified by *in situ* hybridization [24] may be the primary tissues for DWV replication. The observation that DWV actively replicates in multiple tissues involved in egg production, such as the fat body and the ovaries, suggest that DWV could have a major impact on queen fertility. To address this question, we first tried to correlate the DWV titres recorded from a large sampling (130 mated queens) with the queen health status reported by each corresponding beekeeper. No statistically significant correlation could be found between DWV titres and the queen egg-laying performance in these samples, nor with the presence of coloration on the ovaries. We therefore analysed 24 healthy queens of the same age in order to estimate the impact of DWV and VDV-1 infections on the yolk protein precursor (vitellogenin) gene expression as well as the expression of the vitellogenin receptor gene. In honeybees, in addition to being the primary egg yolk precursor, vitellogenin also serve as a precursor of larval food proteins secreted by the hypopharyngeal glands of nurses [49]. Moreover, this protein is involved in immunity and ageing through hormonal regulatory pathways [50] and is therefore a common molecular marker for the overall health of individual bees. As expected, we found a higher expression of vitellogenin in abdomen than in ovaries while the reverse was observed for the vitellogenin receptor. Vitellogenin is produced mainly in the fat body cells and transported to the ovaries where it is absorbed and incorporated in the oocyte until its complete development. However we found no significant relationship between the expression of these genes and the DWV or VDV-1 titres in the different queens, or indeed with the presence of yellow coloration of the ovaries, related to ovariole degeneration. Our data therefore show that DWV and VDV-1 are at best faintly pathogenic for queen honeybees, in the tissues and at the titres encountered in these studies. Their clear association with the degeneration of individual follicles, resulting in the clinical yellowing pathology first described, only seems to affect the functioning of the ovaries as a whole in extreme cases, marked by deeply coloured ovaries. Cases of mild yellowing and follicular degeneration apparently can be compensated for at functional level by the remaining, healthy follicles. This limited pathogenicity of DWV and VDV-1 contrasts with the strong association of DWV incidence in worker bees with winter mortalities of colonies [51]–[53] and the induction of the typical deformed wing pathologies and drastically reduced life expectancy in workers bees [30] as well as sublethal behavioural defects though infection of the brain tissues [54]–[56]. However, in worker bees, most of these symptoms are directly related to quantitative transmission of DWV or VDV-1 by *Varroa destructor* during the pupal phase [40], [57], while the oral and vertical transmission routes, or even Varroa-mediated transmission between adult bees are again minimally pathogenic [30]. One explanation for these discrepancies in pathogenicity may be that transmission by Varroa introduces DWV to certain critical tissues at a particularly sensitive stage of pupal metamorphosis, with the combination of timing, tissue and titre resulting in a lethal pathology, while the reduced titres and tissue restrictions of the other transmission routes result in morphologically and functionally normal bees [58].

The low pathogenicity and high prevalence of DWV and VDV-1 in the host population is a common feature of viruses with long-standing associations with their hosts, particularly when maintained through parent-to-offspring (vertical) transmission [23], [24], [40] and is also found for harmless viruses of other insect species, such as for instance the Sigma virus of drosophila [59], the small RNA virus discovered in *Solenopsis invicta* [60] or the large DNA virus infecting the salivary glands of *Glossinia pallidipes* [61].

### Conclusions

Here we present a newly discovered pathological condition of honeybee queens that in extreme cases can lead to complete ovary impairment. These lesions were found associated with virus particles in massive paracrystalline arrays. Molecular and *in situ* hybridization studies identified these viruses to be most likely DWV and/or VDV-1. However, a large survey of queens showed that in general, DWV and VDV-1 infections had little impact on the health and functional status of the queen, at least in the tissues and at the titres encountered here, suggesting that in most cases the pathology can be compensated for at functional level. This suggests that the accumulation of viral particles in queen ovaries above a certain threshold may lead to the pathological symptoms we observed in some cases, although other factors could also be involved in this phenomenon, including as yet undescribed (viral) pathogens or the accumulation of chemical toxins through the large quantities of food ingested by the queen during its life and the presence of many chemicals residues in pollen [62], [63]. These additional possibilities require further investigation.

## Materials and Methods

### 1. Sample collection

Virgin and mated honeybee queens were received alive from different beekeepers and were immediately dissected in cold PBS buffer under a stereomicroscope or stored at −20°C before RNA extraction.

### 2. cDNA synthesis, PCR and qPCR for the detection and quantification of pathogens in honeybee queens

Total RNA was extracted from individual queens, their ovaries or their abdomens using the Macherey Nagel Nucleospin RNA II® kit, according to manufacturer's instructions. Briefly, each sample was crushed in a 2 ml Eppendorf tube with a 5 mm metal bead in 500 µl denaturing buffer (RA1 from the Nucleospin RNA II® Macherey Nagel Kit) and homogenized for 30 sec with a Tissue Lyzer® apparatus (Qiagen) at maximum speed. Fifty microliters of the homogenate was used for total RNA extraction. About 2 µg of total RNA was retro-transcribed at 25°C for 10 minutes and at 50°C for one hour with the Thermoscript® RT-PCR kit (Invitrogen) using random primers as anchor. A master mix was used for cDNA synthesis. The cDNA was diluted 10-fold in water and stored at −20°C for future qualitative or quantitative PCR assays. An exogenous internal reference RNA consisting of 5×10^7^ copies of Tobacco Mosaic Virus (TMV) was introduced into each sample during total RNA preparation. This allowed us to monitor the efficiency of RNA purification and cDNA synthesis steps, and to reveal the presence of PCR inhibitors in the samples [64].

The qualitative PCR assays for DWV, BQCV, CBPV, ABPV, KBV and SBV were as described previously [46], [64] except that the Eurogentec Gold Taq polymerase and buffer were used. The same reagents, protocols and reaction conditions were used in combination with four newly designed primer pairs for the detection and quantification of four more viruses in the samples: SBPV [65], VDV-1 [29], IAPV [66] and a newly discovered virus (VdMLV; de Miranda, Tournaire, Haddad and Gauthier, unpublished). Assays for *Nosema apis* and *Nosema ceranae* detection were performed as described previously [67].

The quantitative qPCR assays for cDNAs corresponding to the DWV and VDV-1 genomic RNA, and the mRNAs of vitellogenin, vitellogenin receptor [26], and β-actin [27] used the Eurogentec MasterMix Plus for SYBR-Green®, the Applied Biosystems ABI-PRISM 7000 thermocycler and the reaction protocols and conditions described previously [64]. The primer sequences are listed in Table S1, together with the qPCR performance indicators, when appropriate. The qPCR data were converted to the initial amount of each target in the reaction (N_0_) using the LinReg software [68]. The LinReg software calculates the reaction efficiencies (E) for each individual sample and the means of these reaction efficiencies (E) for the different targets, as well as other qPCR performance indicators, are shown in Table S1. The DWV and VDV-1 titres were deduced from N_0_ values calculated from standard curves made of serial dilution series of known amounts of the amplicons [64]. In the analysis of the 24 high-fecundity queens from sampling B (12 with yellow ovaries; 12 with normal ovaries), the DWV and VDV-1 titres were normalized using the amount of β-actin mRNA in each sample as a marker for the quantity and quality of RNA, and are presented as equivalent viral copies per bee or tissue. The amount of vitellogenin and vitellogenin receptor mRNA in each sample was similarly normalized and is expressed relative to the corresponding amount of β-actin mRNA in the sample.

### 3. Histological analysis of honeybee queen tissues

- **Ultrastructural analysis of ovarian follicles.** For transmission electron microscopy (TEM) studies, the germarium part of the ovary was dissected with fine scissors, incubated in 2% glutaraldehyde in a 0.1 M cacodylate buffer (pH 7.4) for 2 hours at 4°C and postfixed in 2% osmium tetroxide for 1 hr at room temperature. The samples were then dehydrated in increasing concentration ethanol baths and embedded in Epon resin for 24 hours. The Epon resin was polymerized for 2 days at 60°C. 0.1 µm cuttings were taken with an ultramicrotome and fixed on copper grids (75–100 mesh). Finally the slices were contrasted with uranyl acetate and lead citrate.
- **Light microscopy observations.** For light microscopy studies, the upper part of the ovaries was fixed in 4% paraformaldehyde in phosphate-buffered saline (PBS) for 24 h at 4°C immediately after dissection. The samples were then dehydrated in increasing ethanol concentration baths, with a final incubation for 24 h in 100% ethanol, before replacing the ethanol with the hydrophilic resin Unicryl (SPI supplies) for 8 hrs at room temperature. The resulting resin blocks were polymerized at 4°C for 3 days, cut in 1 µm slices with a ultramicrotome and stained with 1% Toluidine blue solution.
- ***In situ*** **detection of DWV RNA in queen ovaries.** The *in situ* localization of DWV in the ovaries was done as described previously [24]. Briefly, the ovaries were fixed and dehydrated as for light microscopy, embedded in paraffin blocks and cut in 10 µm slices with a Minot microtome. After rehydration the tissue sections were challenged with the following oligonucleotide probes at a concentration of 200 pmol/ml:
  DWVantisense: 5′-^8917^ TACTGTCGAAACGGTATGGTAAACTGTAC ^8889^-Digoxygenin
  DWVsense: 5′-^8889^ GTACAGTTTACCATACCGTTTCGACAGTA ^8917^-Digoxygenin
  DWVnonsense: 5′-CATGTCAAATGGTATGGCAAAGCTGTCAT -Digoxygenin

The underlined nucleotides in the sense and antisense probes refer to those positions where DWV is different from the closely related VDV-1. These probes hybridize to the negative and positive strand RNA respectively of the DWV/VDV-1 RNA polymerase RNA dependent domain, nucleotides 8889–8917, while the nonsense probe has a similar composition but no affinity to any part of the DWV/VDV-1 genome. The antisense probe was used to detect DWV genomic RNA while the sense and nonsense probes were used in parallel as controls. The hybridized probes were detected by incubating the sections with alkaline phosphatase-conjugated anti-digoxygenin antibody (Roche) and were developed with nitroblue tetrazolium and 5-bromo 4-chloro 3-indolyl phosphate.

- **Propidium iodide staining.** Individual ovarioles were separated in cold PBS and incubated in PBS supplemented with 1 µg/ml of propidium iodide for 15 min at room temperature. The ovarioles were then washed three times in PBS and observed under a fluorescent microscope (Zeiss Axiovert 200M). Alternatively, propidium iodide stained ovaries were embedded in paraffin blocks and cut in 10 µm slices with a Minot microtome. After rehydration the tissue sections were counterstained with DAPI (4′,6-diamidino-2-phenylindole), a fluorescent stain that binds strongly to DNA.

### 4. Cloning and sequencing of viral genomic sequences from queen ovaries

For the cDNA cloning of viral sequences, 12 mated queen ovaries displaying coloration were homogenised in 20 ml of a 10 mM Tris–400 mM NaCl buffer and clarified at 5000 g for 15 min. The supernatant was then ultracentrifuged in a TLA 110 rotor (Beckman) for 1 hr at 110,000 g to concentrate the virions, together with other subcellular particles. The pellet was directly resuspended in Trizol (InVitrogen) and the RNA was extracted according to manufacturer's instructions. About 4 µg of total RNA was used for reverse transcription with the Superscript-III ™ Reverse Transcriptase (InVitrogen), using anchored oligo-dT as the cDNA primer. The cDNA/RNA heteroduplex was digested with RNAse H and converted to double strand DNA, using the partially digested RNA fragments of the heteroduplex as primers for second strand cDNA synthesis [69]. The ds cDNA fragments were 5′-dephosphorylated using shrimp alkaline phosphatase (Promega), a specific requirement for topoisomerase-based ligation into pCR®II-Blunt-TOPO® (InVitrogen). The ligation mixture was transformed into TOP10 Electrocomp™ E. coli cells (InVitrogen) plated on LB agar plates supplemented with 50 µg/ml of kanamycin before screening the colonies for the presence of recombinant plasmids.

### 5. Statistics

When the assumptions of normality for ANOVA were not fulfilled (Shapiro-Wilk and Anderson Darling test) analysis were done using non-parametric statistics. The Kruskal-Wallis test was used to test equality of medians among groups. If the population medians were not equal, a post-hoc Mann-Whitney U-test was applied. Correlations between the data sets were analysed using Pearson correlation. *P*-values lower than 0.05 were considered significant. We also performed Chi-square analysis with Pearson Chi-square or Yates correction to compare discontinuous data, *e.g.* the presence or absence of egg-laying problems, ovary coloration or the different bee viruses (Table 3). All statistical analyses were performed using Systat (Version 12, Systat Software Inc., San Jose, California, USA).

## Supporting Information

Table S1 List of the primers used in this study for classical or quantitative PCR assays. Size: amplicon size. Molarity: primer molarity used in PCR assays. E: qPCR efficiency calculated with the LinReg analysis method. Tm: melting temperature of the amplicon. n.a.: not applicable.
(PDF)

Figure S1 Correlation between DWV equivalent genome copies recorded from abdomen and from ovaries in 24 queens displaying a high fitness.
(TIF)

Figure S2 Expression levels of vitellogenin (Vg) and vitellogenin receptor (Vg-R) mRNA in abdomen and in ovaries of 24 mated queen samples (sampling B). Data were normalized using the β-actin gene.
(TIF)

Figure S3 Additional anomalies and pathologies observed during our queen survey. A, presence of bacterial nodules at the basis of the ovary (arrow). B, melanisation of part of the ovarioles. C, hypoplasia of an ovary. D, presence of fungal infection at the basis of the ovaries. E, sperm clots in the oviducts.
(TIF)
