# Supplementary material for: Viruses Associated with Ovarian Degeneration in Apis mellifera L. Queens
Source: PLoS One. 2011 Jan 25;6(1):e16217. doi: 10.1371/journal.pone.0016217 (PMC3026828; doi:10.1371/journal.pone.0016217)
Supplement: Table S1 — List of the primers used in this study for classical or quantitative PCR assays. Size: amplicon size. Molarity: primer molarity used in PCR assays. E: qPCR efficiency calculated with the LinReg analysis method. Tm: melting temperature of the amplicon. n.a.: not applicable. (PDF) [file pone.0016217.s001.pdf]

SUPPLEMENTARY FILE / Table 4.

	Target	primers	sequence (5' - 3')	size (bp)	Molarity	E	Tm (°C)	References
Qualitative PCR assays	DWV	DWV-F1425<br>DWV-B1806	CGTCGGCCTATCAAAG<br>CTTTTCTAATTCAACTTCACC	417	0.4 µM	n.a.	n.a.	[23]
	VDV-1	VDV1-F1409<br>DWV-B1806	GCCCTGTTCAAGAACATG<br>CTTTTCTAATTCAACTTCACC	430	0.4 µM	n.a.	n.a.	[23]
	SBV	SBV-F<br>SBV-R	GGATGAAAGGAAATTACCAG<br>CCACTAGGTGATCCACACT	426	0.4 µM	n.a.	n.a.	[46]
	SBPV	SBPV-F3177<br>SBPV-R3363	GCGCTTTAGTTCAATTGCC<br>ATTATAGGACGTGAAAATATAC	226	0.4 µM	n.a.	n.a.	[65]
	VdMLV	VdMLV-F<br>VdMLV-R	ATCCCTTTTCAGTTCGCT<br>AGAAGAGACTTCAAGGAC	438	0.4 µM	n.a.	n.a.	
	BQCV	BQCV-F<br>BQCV-R	GTCCAGTGTGATATTGCCAA<br>TCATTAGAAAGCGCCAGACT	550	0.4 µM	n.a.	n.a.	[46]
	ABPV	ABPV-F<br>ABPV-R	CTCAAGTTATACGTAAAATAGCTGGAATT<br>AACCAACCTTGCTTCCCTTTA	646	0.4 µM	n.a.	n.a.	[46]
	KBV	KBV-F<br>KBV-R	GATGAACGTCGACCTATTGA<br>TGTGGGTTGGCTATGAGTCA	414	0.4 µM	n.a.	n.a.	[46]
	IAPV	IAPV-F6627<br>IAPV-R6707	CCATGCCTGGCGATTCAC<br>CTGAATAATACTGTGCGTATC	203	0.4 µM	n.a.	n.a.	
	CBPV	CBPV-F<br>CBPV-R	AGTTGTCATGGTTAACAGGATACGAG<br>TCTAATCTTAGCACGAAAGCCGAG	455	0.4 µM	n.a.	n.a.	[46]

	Target	primers	sequence (5' - 3')	size (bp)	Molarity	E	Tm (°C)	References
Quantitative PCR assays	DWV	DWVq-F<br>DWVq-R	GGATGTTATCTCCTGCGTGGAA<br>CCTCATTAACTGTGTCGTTGATAATTG	69	0.4 µM	1.96	78.5	[64]
	VDV-1	VDV1-F1409<br>DWV-B1806	GCCCTGTTCAAGAACATG<br>CTTTTCTAATTCAACTTCACC	430	0.4 µM	1.78	82.6	[23]
	Vitellogenin mRNA	Vg851F<br>Vg913R	AGATGATGGTCAGCCCTAGACTCT<br>GGTTCATTCTGCTAAGCACCAA	63	0.2 µM	1.84	77.6	
	Vitellogenin receptor mRNA	Vg-R1959F<br>Vg-R2076R	TGAACCTTACGACATTGCCCT<br>TGTGATTTTCGGTCCAAGCCC	117	0.2 µM	1.95	76.7	[26]
	β-Actin mRNA	*A.m.* β-Actin-q92F<br>*A.m.* β -Actin-q157R	CGTTGTCCCGAGGCTCTTT<br>TGTCTCATGAATACCGCAAGCT	66	0.4 µM	1.97	79.6	
