# Supplementary figures and images for: Viruses Associated with Ovarian Degeneration in Apis mellifera L. Queens
Source: PLoS One. 2011 Jan 25;6(1):e16217. doi: 10.1371/journal.pone.0016217 (PMC3026828; doi:10.1371/journal.pone.0016217)

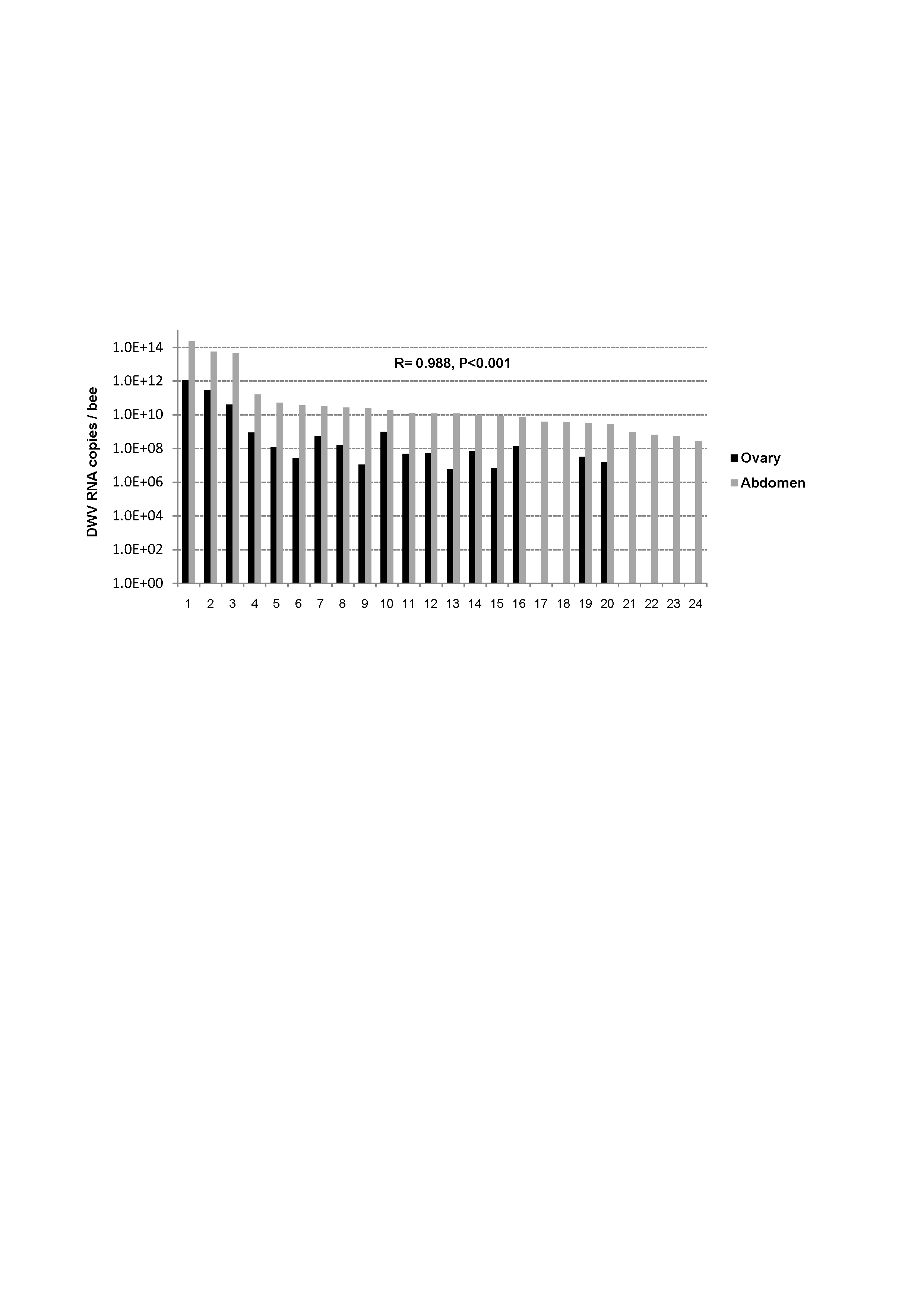

Supplement: Figure S1 — Correlation between DWV equivalent genome copies recorded from abdomen and from ovaries in 24 queens displaying a high fitness. (TIF) [file pone.0016217.s002.tif]

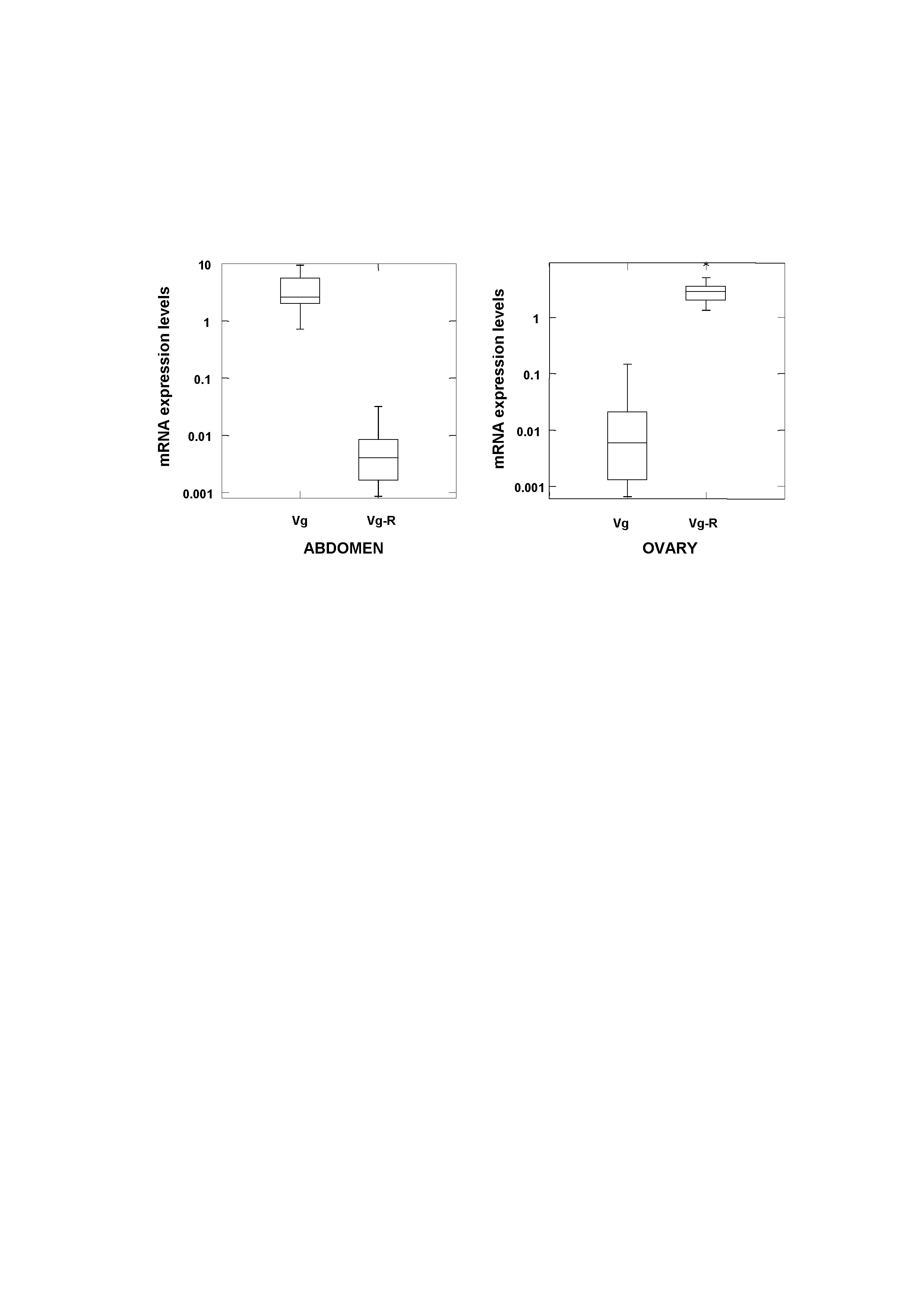

Supplement: Figure S2 — Expression levels of vitellogenin (Vg) and vitellogenin receptor (Vg-R) mRNA in abdomen and in ovaries of 24 mated queen samples (sampling B). Data were normalized using the β-actin gene. (TIF) [file pone.0016217.s003.tif]

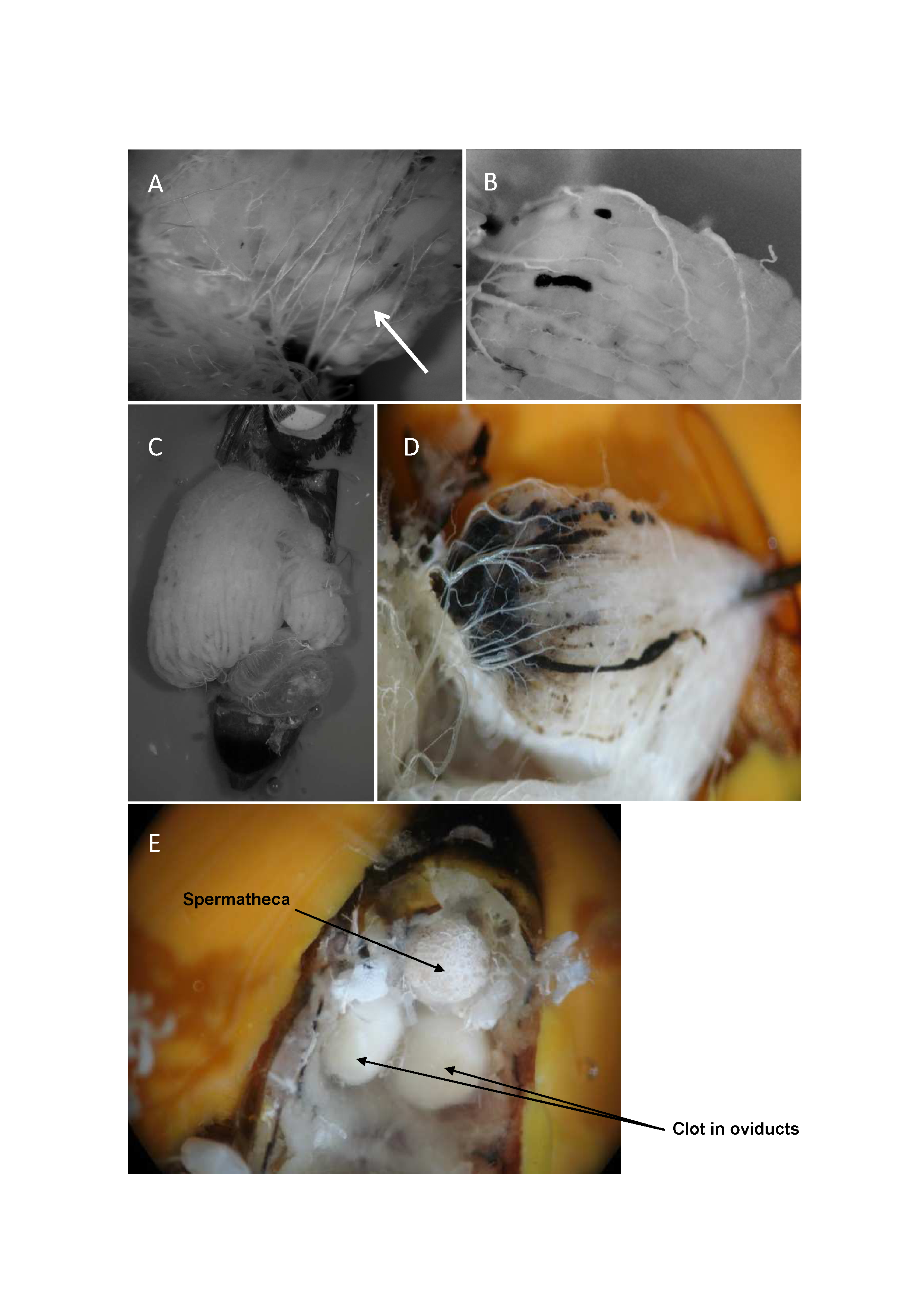

Supplement: Figure S3 — Additional anomalies and pathologies observed during our queen survey. A, presence of bacterial nodules at the basis of the ovary (arrow). B, melanisation of part of the ovarioles. C, hypoplasia of an ovary. D, presence of fungal infection at the basis of the ovaries. E, sperm clots in the oviducts. (TIF) [file pone.0016217.s004.tif]
